# Diverse effect of BMP-2 homodimer on mesenchymal progenitors of different origin

**DOI:** 10.1007/s13577-018-0202-5

**Published:** 2018-02-13

**Authors:** Edit Hrubi, László Imre, Agnieszka Robaszkiewicz, László Virág, Farkas Kerényi, Krisztina Nagy, Gábor Varga, Attila Jenei, Csaba Hegedüs

**Affiliations:** 1 Faculty of Dentistry, University of Debrecen, Nagyerdei krt. 98, Debrecen, 4012 Hungary; 2 Department of Biophysics and Cell Biology, Faculty of Medicine, University of Debrecen, Egyetem tér 1, Debrecen, 4032 Hungary; 3 Department of General Biophysics, Institute of Biophysics, Faculty of Biology and Environmental Protection, University of Lodz, Pomorska 141/143, 90-236 Lodz, Poland; 4 Department of Medical Chemistry, Faculty of Medicine, University of Debrecen, Egyetem tér 1, Debrecen, 4032 Hungary; 5 Department of Oral Biology, Semmelweis University, Nagyvárad tér 4, Budapest, 1089 Hungary

**Keywords:** Stem cells, Osteogenic differentiation, Alizarin red, Alkaline phosphatase, Growth factor

## Abstract

Bone morphogenetic protein-2 (BMP-2), is a potential factor to enhance osseointegration of dental implants. However, the appropriate cellular system to investigate the osteogenic effect of BMP-2 in vitro in a standardized manner still needs to be defined. The aim of this study was to examine the effect of BMP-2 on the cell proliferation and osteogenic differentiation of human osteogenic progenitors of various origins: dental pulp stem cells (DPSC), human osteosarcoma cell line (Saos-2) and human embryonic palatal mesenchymal cell line (HEPM). For induction of osteogenic differentiation, cell culture medium was supplemented with BMP-2 homodimer alone or in combination with conventionally used differentiation inducing agents. Differentiation was monitored for 6–18 days. To assess differentiation, proliferation rate, alkaline phosphatase activity, calcium deposition and the expression level of osteogenic differentiation marker genes (Runx2, BMP-2) were measured. BMP-2 inhibited cell proliferation in a concentration and time-dependent manner. In a concentration which caused maximal cell proliferation, BMP-2 did not induce osteogenic differentiation in any of the tested systems. However, it had a synergistic effect with the osteoinductive medium in both DPSC and Saos-2, but not in HEPM cells. We also found that the differentiation process was faster in Saos-2 than in DPSCs. Osteogenic differentiation could not be induced in the osteoblast progenitor HEPM cells. Our data suggest that in a concentration that inhibits proliferation the differentiation inducing effect of BMP-2 is evident only in the presence of permissive osteoinductive components. β-glycerophosphate, was identified interacting with BMP-2 in a synergistic manner.

## Introduction

One of the most important issues in dental implantology is to conduce osteogenic integration of dental implants by the modification of titanium surface. Many efforts have been made aiming to enhance cell adhesion and bone formation by several molecules linked to titanium. Different bioactive organic macromolecules could be suitable for modification of the surface of dental implants such as BMP-2 and BMP-7 approved by the American Food and Drug Administration (FDA) to use in the clinical practice: [1]. To reproducibly test the osteogenic effects of such compounds, reliable in vitro test systems are needed.

In the present study, the BMP-2 homodimer protein was selected to use, which is known to initiate osteogenic differentiation and bone formation both in vitro [2–4] and in vivo [2, 4, 5]. BMP-2 belongs to the BMP subgroup of the transforming growth factor-β (TGF-β) protein superfamily involved in the regulation of multiple organogenic developmental processes including bone formation and skeletogenesis [6, 7]. In a comparative analysis, 14 members of the BMP protein family were studied to identify factors with the most potent osteoinductive activity. It turned out that BMP-2, BMP-6 and BMP-9 showed the most potent osteogenic activity [8]. The functional form of BMP-2 is a homodimer which is the ligand of the cell surface BMP receptors (BMPRI, BMPRII). Binding of the BMP-2 homodimer activates intracellular signal transduction through the SMAD or MAPK pathways [9] which can interact with other signaling pathways through FGF, Hedgehog and Wnt proteins regulating the expression of several transcription factors such as Sox 9, Cbfa1 (Runx2) and Msx [10] involved in osteogenic differentiation and bone formation.

Here we report a comparative study investigating the effect of recombinant BMP-2 homodimer proteins on osteogenic differentiation of human dental pulp stem cells (DPSC) isolated from the pulp tissue of healthy human wisdom teeth and two commonly used preosteoblast cell lines, namely Saos-2 osteosarcoma cells and human embryonic palatal mesenchymal preosteoblast cells (HEPM). Most studies investigating the effect of BMP-2 involve only one cell type. In contrast to the shortcomings due to the application of a single cell type, multiple cell types offer more precise and valid analysis. Published data have shown that the effect of BMP-2 depends both on the environment and the cell type [11]. Furthermore, the effect of BMP-2 on DPSCs has been poorly studied. Therefore, our aim was to determine the effective concentration of BMP-2, to study its effect on DPSCs in comparison with two other cell lines, commonly used in osteogenic differentiation experiments and to analyze BMP-2 applied alone and in different molecular environments containing agents conventionally used to induce osteogenic differentiation.

## Materials and methods

### Cell growth and osteogenic differentiation

Human dental pulp stem cells (DPSCs) were isolated from the pulp tissue of healthy human wisdom teeth as it was described previously [12], and were sorted for STRO-1 cell surface marker [13] (patient declaration of agreement No. F0102/1ST). Human embryonic palatal mesenchymal cells (HEPM, ATCC No.: CRL-1486) Saos-2 osteosarcoma cells (ATCC No.: HTB-85) and STRO-1 positive DPSCs were cultured in Eagle’s Minimum Essential Medium (EMEM, Sigma Aldrich, M5650), Dulbecco’s Modified Eagle’s Medium (DMEM, Sigma Aldrich, D6046) and Alpha modified Minimum Essential Medium (αMEM, Sigma Aldrich, M4526), respectively, supplemented with 10% FBS (Sigma Aldrich, F9665), 100 units/ml penicillin and 100 mg/ml streptomycin (Sigma Aldrich, P0781), and 1% GlutaMAX (Life technologies, 10567014) at 37 °C, 5% CO_2_ in a humidified atmosphere. These culture media are further indicated on the figures and in the text as control media (CM).

Osteoinductive medium (OIM) was prepared by supplementing CM with 10 mM β-glycerophosphate (Sigma Aldrich, G9891), 50 µg/ml ascorbic acid (Sigma Aldrich, 1043003), 0.1 µM dexamethasone (Sigma Aldrich, D4902) and 50 nM vitamin D3 (Sigma Aldrich, 740292).

Media indicated on the figures as β-GLY, AA, DEX and D3 VIT were prepared by supplementing CM with 10 mM β-glycerophosphate (β-GLY), 50 µg/ml ascorbic acid (AA), 0.1 µM dexamethasone (DEX) or 50 nM vitamin D3 (D3 VIT), respectively.

Media indicated on the figures as CM + BMP-2, OIM + BMP-2 and β-GLY + BMP-2 were prepared by supplementing CM, OIM and β-GLY media with BMP-2 (Antibodies-online GmbH, Z00327) in a final concentration of 0.1 µg/ml.

BMP-2 stock solution was prepared from lyophilized recombinant human BMP-2 according to manufacturer’s instructions in a final concentration of 1 mg/ml and aliquots were stored at − 80 °C until use.

### Alamar blue assay

**Fig. 1:**
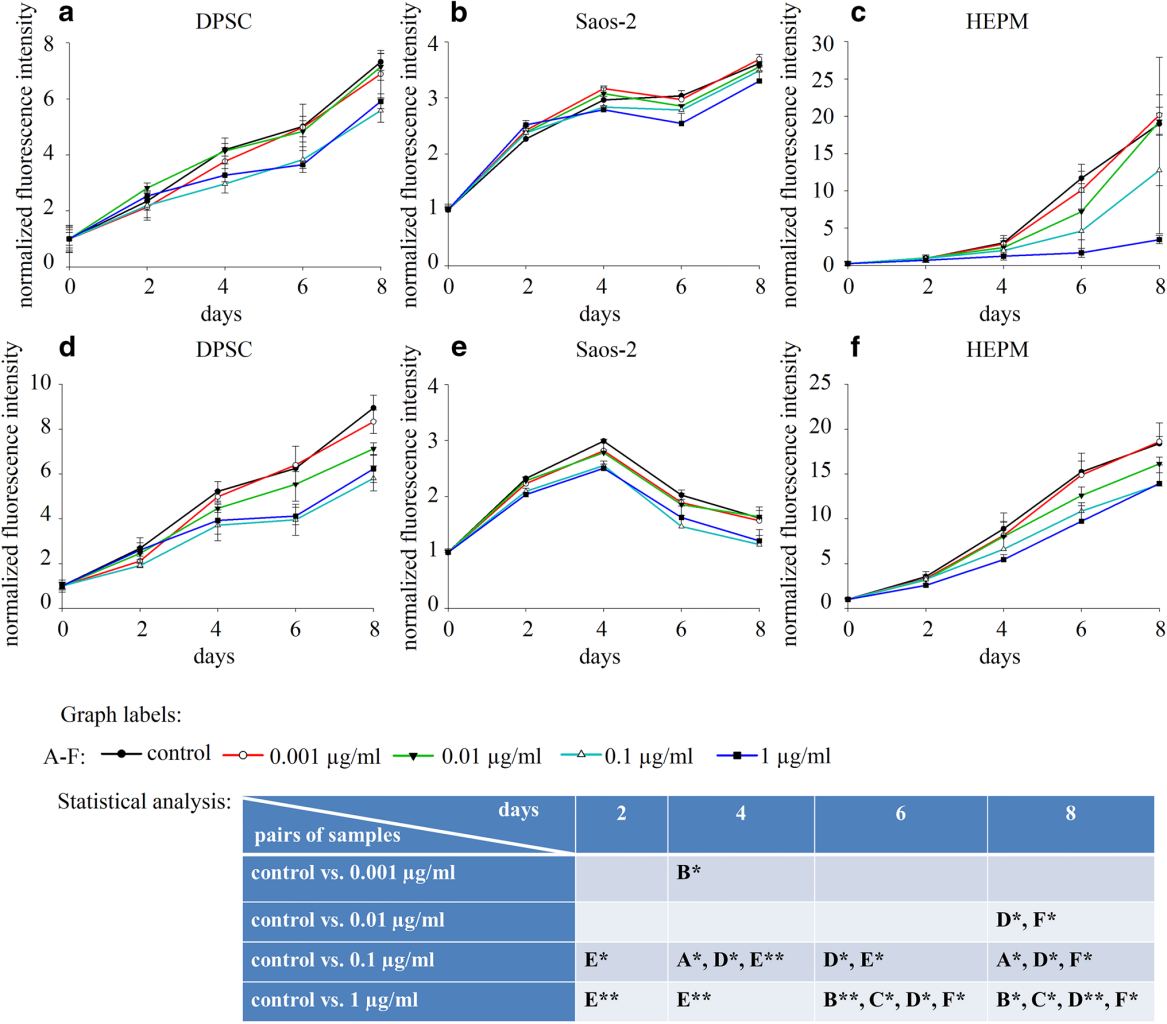
Measurements of cell proliferation by alamar blue assay. DPSC (**a**), Saos-2 (**b**) and HEPM (**c**) cells were examined in control medium (CM) supplemented with different concentrations of BMP-2. Experiments were carried out using OIM medium supplemented with the same concentration series of BMP-2 on DPSC (**d**), Saos-2 (**e**) and HEPM (**f**) cells. Error bars represent standard deviation calculated from three parallel measurements. Statistical analysis was performed using ANOVA followed by Bonferroni statistical test. *P* < 0.05 and *P* < 0.005 values were labeled on the figure as “*” and “**”, respectively

**Fig. 2:**
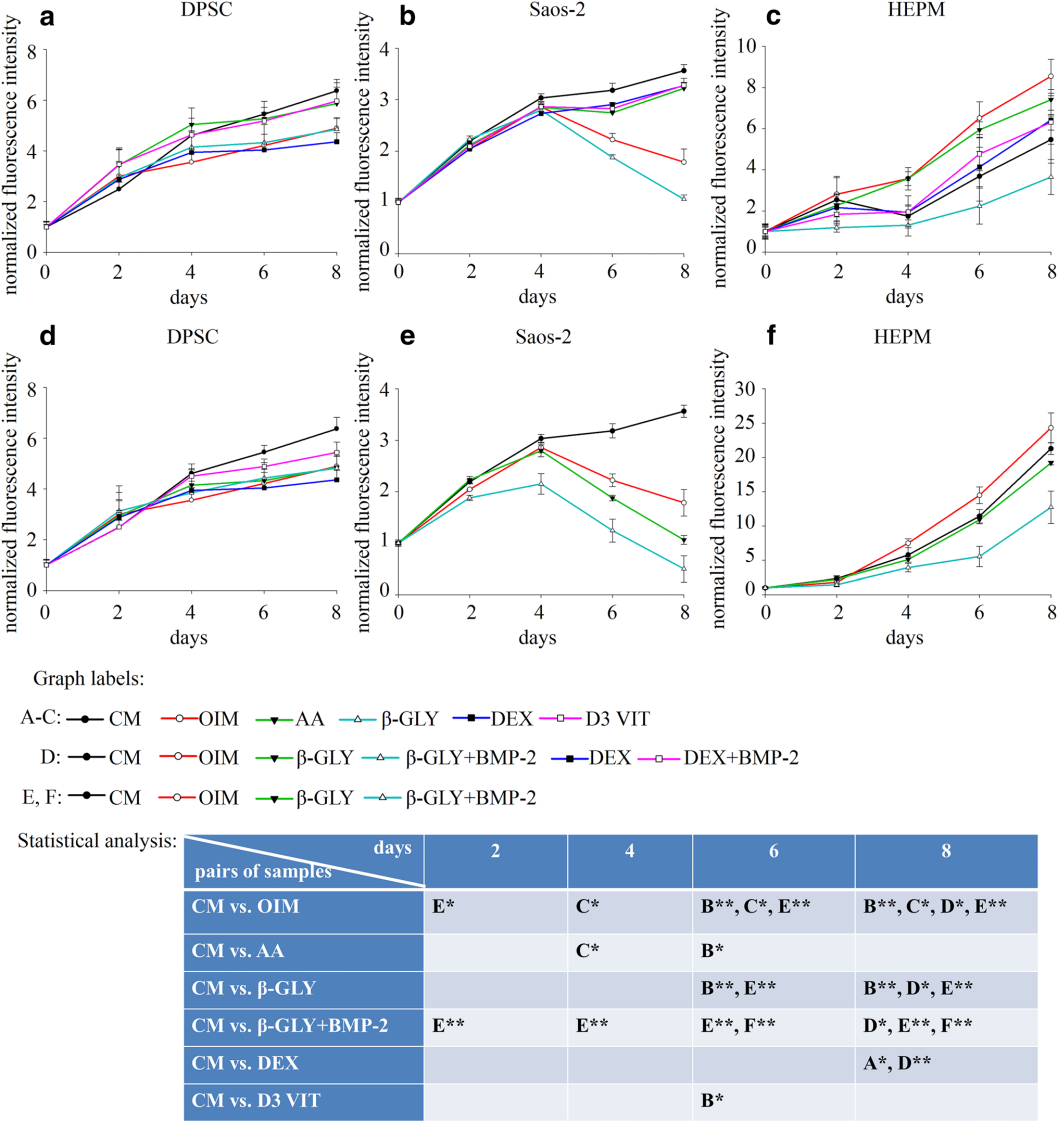
Measurements of cell proliferation by alamar blue assay on DPSC (**a**), Saos-2 (**b**) and HEPM (**c**) cells cultured in control medium (CM) supplemented with different components of the OIM medium such as: ascorbic acid (AA), β-glycerophosphate (β-GLY), dexamethasone (DEX), vitamin D3 (D3 VIT). Those components which decreased proliferation (β-glycerophosphate and dexamethasone) were combined with BMP-2 and were examined on DPSC (**d**), Saos-2 (**e**) and HEPM (**f**) cells. Error bars represent standard deviation calculated from three parallel measurements. Statistical analysis was performed using ANOVA followed by Bonferroni statistical test. *P* < 0.05 or *P* < 0.005 values were labeled on the figure as “*” and “**”, respectively

Into each well of a 96 well cell culture plate (VWR, 89093-608), 1000 cells in the case of HEPM and Saos-2 and 7000 cells in the case of DPSC were seeded in 200 µl CM. Cells were allowed to attach overnight, then CM was removed (day 0), cells were washed with 200 µl colourless DMEM (Sigma Aldrich, D5921) and were incubated in 200 µl alamar blue reagent (Life Technologies, DAL 1100) diluted 1:10 in colourless DMEM. Incubation was for 3 h in the case of HEPM and Saos-2 cells and 2 h in the case of DPSC at 37 °C in 5% CO_2_. Alamar blue assay was previously optimized for each cell type (data not shown). After incubation, fluorescence of the reduced alamar blue was measured by Hidex Sense Microplate reader using 535 nm excitation light and 595 nm emission filter. After the measurement, alamar blue was changed to the appropriate media (Figs. 1, 2) and culturing was countinued. Measurements were repeated at every second day during an 8-day long interval. Fluorescence intensities were normalized to the fluorescence measured at day 0.

### Measurement of calcium deposition

**Fig. 3:**
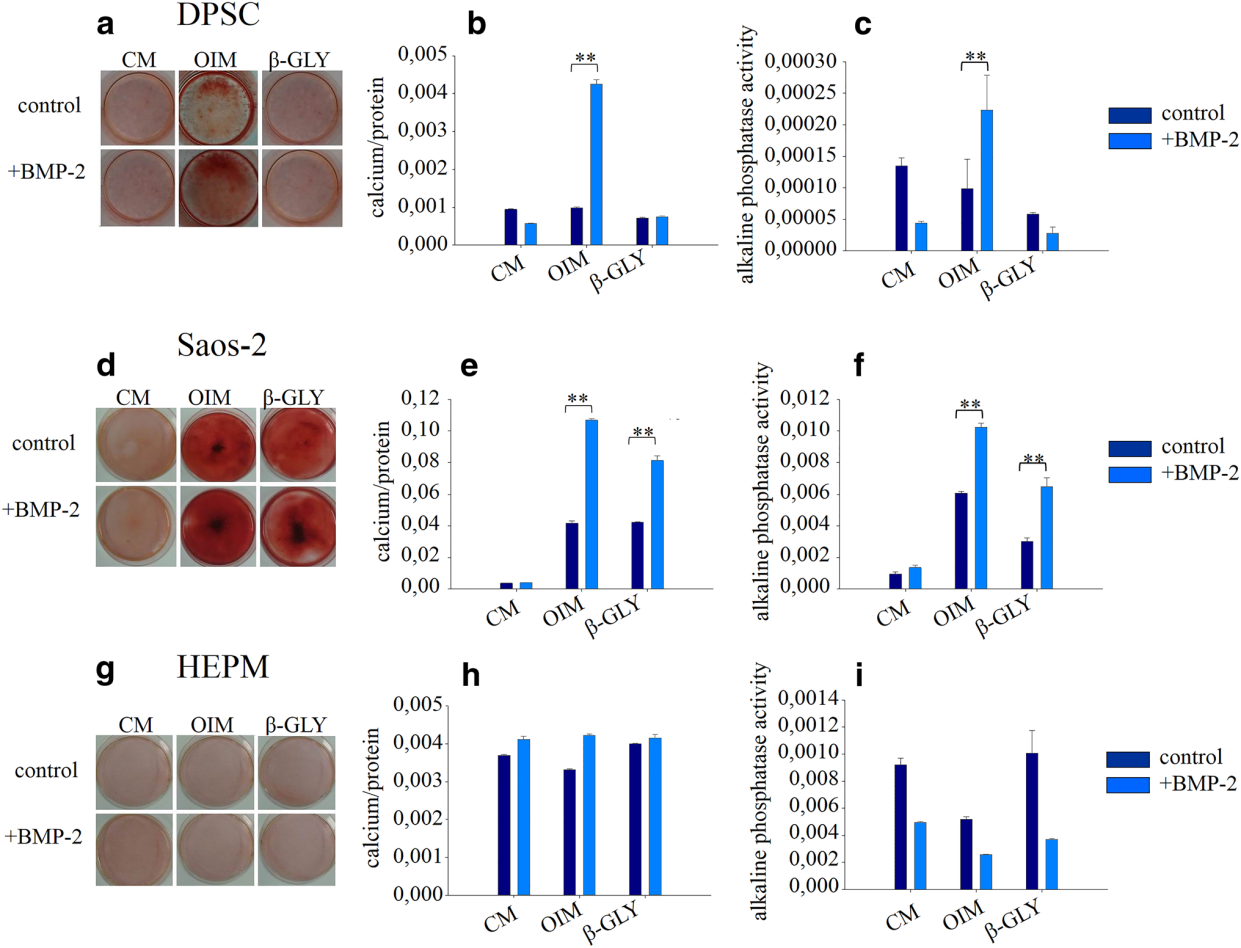
Mineralization of DPSC (**a**) Saos-2 (**d**), and HEPM (**g**) cells were visualized by Alizarin Red S staining at the 6th day of differentiation. Calcium bound dye was extracted and quantitatively analyzed by spectrofluorimeter in DPSC (**b**) Saos-2 (**e**), and HEPM (**h**) cells. From parallel samples alkaline phosphatase activity was also measured in DPSC (**c**) Saos-2 (**f**), and HEPM (**i**) cells. Error bars represent standard deviation calculated from three parallel measurements. Statistical analysis was performed using ANOVA followed by Bonferroni statistical test. *P* < 0.05 or *P* < 0.005 values were labeled on the figures as “*” and “**”, respectively

**Fig. 4:**
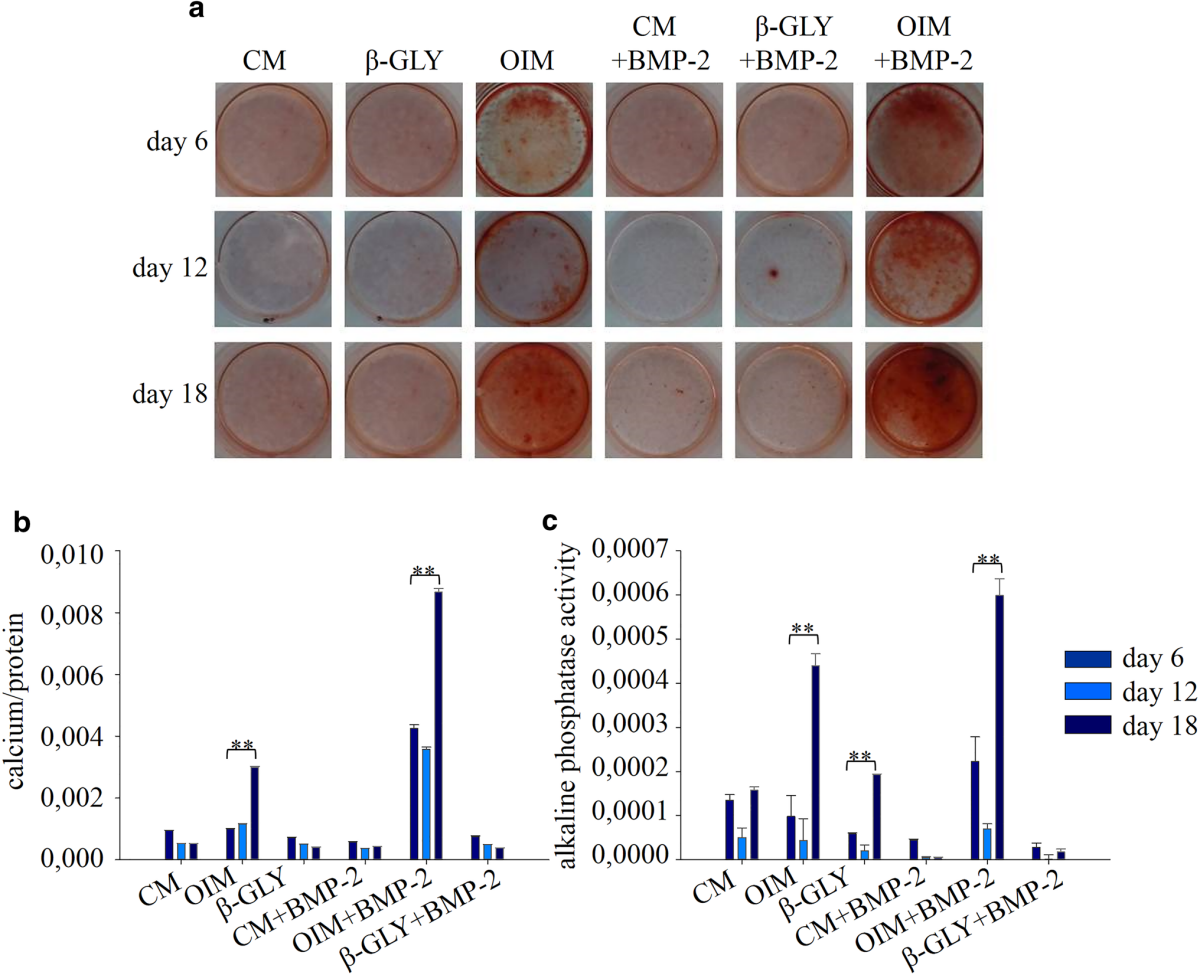
Visualization by Alizarin Red staining (**a**) and quantification of calcium deposition (**b**), as well as alkaline phosphatase activity (**c**) of DPSC cells at the 6th, 12th and 18th days of differentiation. Error bars represent standard deviation calculated from three parallel measurements. Statistical analysis was performed using ANOVA followed by Bonferroni statistical test. *P* < 0.05 or *P* < 0.005 values were labeled on the figure as “*” and “**”, respectively

Into each well of a 6 well cell culture plate, 300,000 cells were seeded in 2 ml CM. Cells were allowed to attach overnight, then culture medium was changed (day 0) to the appropriate media involved in the experiments (see Figs. 3, 4). Media were changed every second day. After 6, 12 or 18 days media were removed, cells were washed two times with 2 ml 1 × PBS (Sigma Aldrich, P5493) and were dehydrated with 2 ml ice cold methanol (Sigma Aldrich, 322415) for 30 min. After removal of the methanol, samples were dried out for 5 min at room temperature and were stained with 2% (w/v) Alizarin Red S solution (Sigma Aldrich, A5533) adjusted to pH 4.27 in the case of HEPM and Saos-2 cells or adjusted to pH 7 in the case of DPSC (Alizarin Red S adjusted to pH 4.27 did not stain DPSCs nicely). For quantification of the staining, Alizarin Red S—calcium complexes were extracted with 10% cetylpyridinium chloride (Sigma Aldrich, C0732) diluted in 10 mM sodium phosphate buffer adjusted to pH 7 (Sigma Aldrich, P5244) and absorbance was measured by a Hidex Sense Microplate reader at 570 nm. For standardization, protein concentration of cell lysates was determined with Pierce BCA Protein Assay (Thermo Scientific, 23227) (according to instruction provided by manufacturer) from parallel samples that were not stained with Alizarin Red S. Quantified calcium deposition was expressed as A_570nm_/µg protein.

### Alkaline phosphatase (ALP) activity

Into each well of a 6 well cell culture plate, 300,000 cells were seeded in 2 ml CM. Cells were allowed to adhere overnight, then culture medium was changed (day 0) to the appropriate media involved in the experiments (Figs. 3, 4). Media were changed every second day. After 6, 12 or 18 days, media were removed and cells were washed two times with 2 ml 1 × PBS. Cells were lysed in lysis buffer [10 mM Tris–HCl pH: 7.4, 100 mM NaCl, 1 mM EDTA, 1% Triton X-100, 1% protease inhibitor cocktail (PIC), 1% phenylmethylsulfonyl fluoride (PMSF)] and scraped lysate was transferred into Eppendorf tubes. After centrifugation (10,000*g*, 10 min, 4 °C), the supernatant was used for the determination of enzyme activity as well as protein concentration (Pierce BCA Protein Assay, Thermo Scientific, 23227). Measurement of alkaline phosphatase activity was conducted 6 min after mixing cell lysates with the enzyme–substrate, 0.1% *p*-nitrophenyl phosphate (Sigma Aldrich, N7653; in 0.1 M glycine, 1 mM MgCl_2_, ZnCl_2_, pH 10.4) at 405 nm by a Hidex Sense Microplate reader. ALP activity was normalized to protein concentration and expressed as A_405nm_/µg protein.

### Total RNA extraction, reverse transcription, and real-time PCR analysis

**Fig. 5:**
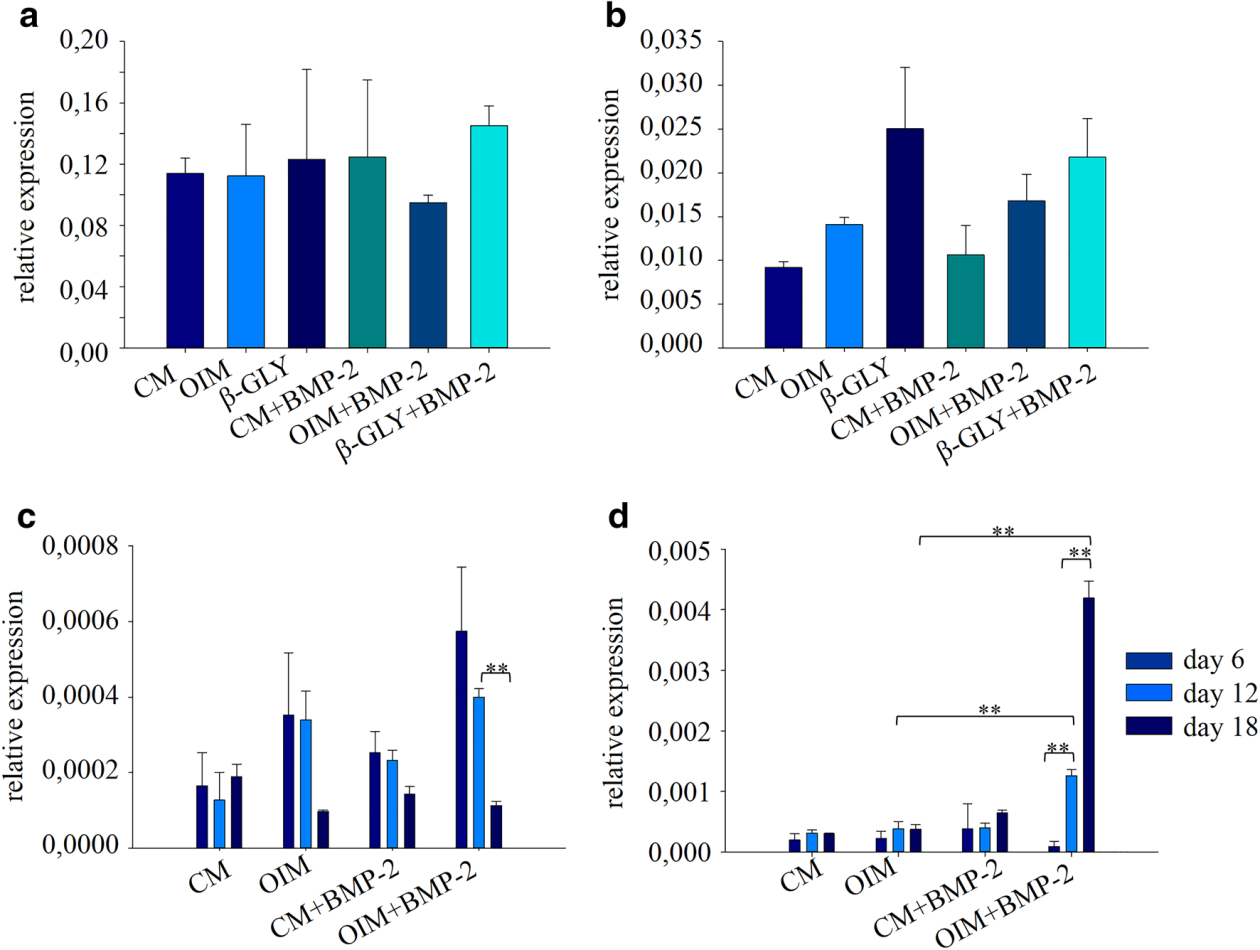
Expression level of Runx2 (**a**) and BMP-2 (**b**) genes were quantitatively analyzed in Saos-2 cells cultured for 4 days at different conditions found to enhance mineralization and alkaline phosphatase activity. Runx2 (**c**) and BMP-2 (**d**) gene expression analysis were performed at the 6th, 12th and 18th days of differentiation in DPSCs. Error bars represent standard deviation calculated from three parallel measurements. Statistical analysis was performed using ANOVA followed by Bonferroni statistical test. *P* < 0.05 or *P* < 0.005 values were labeled on the figure as “*” and “**”, respectively

Into each well of a 6 well cell culture plate, 300,000 cells were seeded in 2 ml CM. Cells were attached overnight, then culture medium was changed (day 0) to the appropriate media involved in the experiments (Fig. 5). Media were changed every second day. After 4 days in the case of Saos-2 or after 6, 12 or 18 days in the case of DPSCs media were removed and cells were washed two times with 2 ml 1 × PBS. Cell lysis, total RNA extraction, real-time quantitative PCR and calculation of relative expressions were performed as described: [14]. Expression levels of the genes analyzed [TaqMan gene expression assays: Runx2 (Applied Biosystems, Hs00231692_m1), BMP-2 (Applied Biosystems, Hs00154192_m1)] were normalized to the reference house keeping gene GAPDH (glycerol-aldehyde-3-phosphate dehydrogenase) in each sample.

### Statistical analysis

Error bars on the figures represent the standard deviation calculated from three parallel measurements. Statistical analysis was performed using ANOVA, Bonferroni statistical test. *P* < 0.05 or *P* < 0.005 values were labeled on the figures as “*” and “**”, respectively.

## Results

### BMP-2 inhibits cell proliferation

The effect of BMP-2 on cell proliferation was studied for 8 days on DPSC, Saos-2 and HEPM cells treated with a concentration series of BMP-2 diluted in CM (Fig. 1a–c) or in OIM (Fig. 1d–f). In CM all three types of cells proliferated during the whole study independently from the presence or absence of BMP-2. However, when supplementing CM with BMP-2, the rate of cell growth was decreased in a concentration dependent manner. The same phenomenon was observed in OIM, except for Saos-2. In OIM the proliferation of Saos-2 cells stopped at day 4, thereafter the number of cells (as determined with alamar blue assay) decreased over time at day 6 and 8., This is in line with findings of others [14].

Investigating the components of the OIM, we found that only β-GLY had an effect on all the three cell lines decreasing cell growth. DEX alone had inhibitory effect but only on DPSC cells (Fig. 2a–c). Moreover, a synergistic effect could be observed between β-GLY and BMP-2 in the case of Saos-2 and HEPM (Fig. 2d, e). The combination of BMP-2 with β-GLY or with DEX did not result in such interaction in DPSCs (Fig. 2f).

### BMP-2 increases mineralization and alkaline phosphatase activity in a cell type and molecular environment dependent manner

Mineralization was measured in CM, OIM and CM supplemented with β-GLY after 6 days of incubation. From the components of the OIM medium, only β-GLY was investigated since it was the only agent that showed similar effect on all cell types in the proliferation assays. After 6 days, staining could be seen in Alizarin Red stained DPSC cells cultured in OIM, but it could not be quantified by spectrofluorimetry. The alkaline phosphatase activity did not change either compared to control cells grown in CM. A significant increase could be observed in the mineralization when OIM was supplemented with BMP-2 which was verified by quantitative measurement (Fig. 3a, b) as well. In parallel with Ca deposition, an increased alkaline phosphatase activity was also measured (Fig. 3c). In the case of Saos-2 cells significant calcium deposition could be observed not only in OIM, but also in β-GLY (Fig. 3d, e). The alkaline phosphatase activity correlated well with the level of mineralization (Fig. 3f). When combining BMP-2 with OIM or with β-GLY, synergistic effects were observed with both media (Fig. 3d–f). In contrast to DPSC and Saos-2 cultures, neither mineralization, nor increased alkaline phosphatase activity were detected in HEPM cells (Fig. 3g–i).

The differentiation was followed not only for 6 days, but also for 12 and 18 days. This longer study could not be performed on Saos-2 cells cultured in OIM since after the 6th day the number of live cells decreased (Fig. 1e). Thus, at day 8 not enough cell lysates could be collected to perform accurate measurements. In contrast to Saos-2, DPSC and HEPM cells could be cultured and differentiated for more than 6 days. In DPSC cells cultured in OIM independently of the presence or absence of BMP-2, no further mineralization was measured and alkaline phosphatase activity seemed to be decreased at day 12 compared to day 6. However, this difference was not statistically significant. When supplementing OIM medium with BMP-2, a synergistic effect could be detected. The effect of OIM medium was more pronounced when the differentiation was performed for 18 days and the synergistic effect between BMP-2 and OIM still existed (Fig. 4a–c). Longer differentiation was also performed on HEPM cells where no detectable changes were observed (data not shown).

### BMP-2 upregulates osteogenic marker genes in a cell type and molecular environment dependent manner

The expression levels of early marker genes Runx2 and BMP-2 was investigated using reverse transcription quantitative real-time PCR. In these experiments we only used those treatments which were found to enhance mineralization and increase alkaline phosphatase activity. Saos-2 cells were grown for 4 days in CM, OIM or β-GLY media in the presence or absence of BMP-2. For gene expression analysis 4-day long differentiation was selected according to published data [14]. In the case of Saos-2 the expression level of Runx2 did not change at day four in response to any of the treatments. However, the level of endogenous BMP-2 significantly increased in cells treated with β-GLY and moderately increased in OIM. BMP-2 had synergistic effects in both cases (Fig. 5a, b).

DPSC cells could be differentiated longer than Saos-2. Therefore, gene expression levels could be analyzed at the 6th, 12th and 18th days of differentiation, which were the same time points as in mineralization and alkaline phosphatase activity measurements. Cells were grown in CM, OIM, CM + BMP-2 and OIM + BMP-2. At day 6 the expression level of Runx2 was the highest as a result of OIM + BMP-2 treatment and it was statistically significant compared to cells in CM. Moderate and minor, statistically not significant increases of expression level were measured in OIM in CM + BMP-2, respectively. From day 6, Runx2 expression gradually decreased over time as detected at days 12 and 18 (Fig. 5c). In contrast, the expression level of BMP-2 gradually increased over time from day 6 to day 18 in response to OIM + BMP-2 (Fig. 5d), showing reverse correlation with Runx2. In OIM and CM + BMP-2 no changes were detected in BMP-2 expression compared to control CM.

## Discussion

BMP-2 is known to enhance osteogenic differentiation. However, treating cells with BMP-2 alone may result in various outcomes depending on the cell type and the osteoinductive molecules (molecular environment) [15–17].

In the present study BMP-2 was examined alone or in combination with different compounds known to initiate osteogenic differentiation involving different cell types, namely DPSC, Saos-2 and HEPM. BMP-2 inhibited the proliferation of all the three cell types in a concentration dependent manner both alone, in CM and in combination with a mixture of osteoinductive agents. OIM is known to initiate osteogenic differentiation and is commonly used in such experiments. Investigating the contribution of its components to osteogenic differentiation we found that only β-GLY inhibited cell proliferation in all three cell types. When cells were treated with BMP-2 in combination with β-GLY, synergistic effects were found on the inhibition of proliferation in HEPM and Saos-2 cells. According to published data, the decreased proliferation rate may be related to the differentiation process [8, 18–20].

Further investigating its osteogenic differentiation effect, BMP-2 alone had no effect on alkaline phosphatase activity. However, when combining BMP-2 with β-GLY or with OIM medium, synergistic effects could be detected in Saos-2 cells. An increased level of alkaline phosphatase activity was also measured in DPSCs as a result of a synergistic effect, but only when BMP-2 was combined with OIM. β-GLY is the substrate of the alkaline phosphatase enzyme known to have increased activity in mature osteoblasts [21–23]. Hydrolysis of β-GLY results in the extracellular accumulation of inorganic phosphate that can react spontaneously with the Ca^2+^ ions of the culture medium forming solid crystals which precipitate in the extracellular matrix [22–24]. In our differentiation experiments when increased ALP activity was detected, the amount of deposited crystals showed positive correlation with ALP in line with expectations.

When studying osteogenic marker genes, OIM increased the expression of Runx2 which was further increased by BMP-2 in DPSCs differentiated for 6 days. From the components of OIM, DEX, AA and β-GLY can increase Runx2 expression through signaling pathways involving TAZ, MKP-1 and FHL-2 proteins or phosphorylation of ERK1/2. [25]. When differentiating DPSCs for more than 6 days, Runx2 level decreased gradually over time as the maturation progressed which is in line with published data [26]. While Runx2 expression is low in committed osteoprogenitors, it becomes elevated during differentiation in preosteoblasts and immature osteoblasts, then it decreases again in mature osteoblast and osteocytes [27, 28].

In Saos-2 cells, differentiation appears to be terminated earlier, than in DPSCs as suggested by our proliferation measurements and also by published data [14]. Since no differences in Runx2 expression could be detected between control and differentiated samples, it can be hypothesized that the mRNA level of Runx2 has already decreased at day four as a consequence of the shorter differentiation time. According to recently published data, another explanation also arises. In Saos-2 cells, p53 tumor suppressor, which indirectly inhibits Runx2 expression through micro RNA-34, is downregulated. Thus, decreased level of p53 may result in elevated level of Runx2 [29]. Therefore, high Runx2 expression may not be increased further in Saos-2 cells exposed to osteogenic stimuli.

The expression level of endogenous BMP-2 correlated well with the increased activity of the alkaline phosphatase enzyme. This can be explained by the increased concentration of extracellular inorganic phosphate known to be able to enter into the cell via phosphate transporters [24]. In turn inorganic phosphate can trigger signaling through ERK and cAMP/protein kinase and activation of these signaling pathways increases the expression of BMP-2 [25]. This is in line with the increasing level of endogenous BMP-2 both in DPSC and Saos-2 cells.

Based on our results it can be concluded that BMP-2 alone is not a sufficient signal to induce the maturation of osteoblast progenitors examined. However, in an appropriate molecular environment permissive for osteogenic differentiation, containing molecules such as β-GLY, BMP-2 can act in a synergistic manner in the case of DPSC and Saos-2 cells.
